# Euthanasia and Pain in Canine Patients with Terminal and Chronic-Degenerative Diseases: Ethical and Legal Aspects

**DOI:** 10.3390/ani13071265

**Published:** 2023-04-06

**Authors:** Daniel Mota-Rojas, Adriana Domínguez-Oliva, Julio Martínez-Burnes, Alejandro Casas-Alvarado, Ismael Hernández-Ávalos

**Affiliations:** 1 Neurophysiology, Behavior and Animal Welfare Assessment, Universidad Autónoma Metropolitana (UAM), Mexico City 04960, Mexico; 2 Facultad de Medicina Veterinaria y Zootecnia, Universidad Autónoma de Tamaulipas, Victoria City 87000, Mexico; 3 Clinical Pharmacology and Veterinary Anesthesia, Facultad de Estudios Superiores Cuautitlán, Universidad Nacional Autónoma de México (UNAM), Cuautitlán 54714, Mexico

**Keywords:** companion animals, euthanasia, chronic disease, chronic pain

## Abstract

**Simple Summary:**

Euthanasia is a common procedure performed by veterinary professionals, which carries important ethical, moral, and legal challenges. In the case of dogs with chronic and terminal illnesses, the decision is highly influenced by owner-related perspectives, financial constraints, caregiver burden, and emotional attachment. Likewise, although the current legal framework in different countries states that euthanasia should be applied as a last alternative in those animals with incurable pain and suffering, the veterinarian must comply with both legal and social responsibilities. The present review explores the ethical and legal implications of euthanasia in canine patients, highlighting the importance of effective communication, ethical knowledge, and consideration of euthanasia as a multimodal resolution.

**Abstract:**

Euthanasia is commonly performed in veterinary medicine to humanely induce the death of an animal when its quality of life is affected by pain or chronic degenerative diseases. The choice of euthanasia is a bilateral decision that represents a challenge for both the veterinarian and the owner of the animal due to the close emotional human–animal bond. Currently, there is legislation that can orient veterinarians concerning euthanasia and the causes that would justify this resolution. However, it is still controversial, and deciding it as the last available resort requires considering it from a medical, legal, and moral perspective. Therefore, this review aims to explore the ethical and legal implications of euthanasia in canine patients. It will analyze the reason that can justify euthanasia in animals with pain or terminal and chronic degenerative diseases, highlighting the importance of effective communication, ethical knowledge, and consideration of euthanasia as a multimodal resolution.

## 1. Introduction

Euthanasia comes from the Greek words eu (good) and thanatos (death), meaning “the act of painlessly inducing a humane death to an animal” [1]. Within the legal framework, euthanasia is considered a method that rapidly induces loss of consciousness and subsequent death without pain or distress [2]. In veterinary medicine, it is a common practice performed in older animals or those affected by terminal and chronic illnesses [3,4].

Despite the professional qualification of veterinarians to use several euthanasic agents depending on the species, and make recommendations about the outcome depending on the health of companion animals, it is considered a controversial and stressful event for both the owner and the clinician [5,6]. Adopting an ethical perspective is one of the proposed approaches to objectively decide whether euthanasia is the best option [7]. However, the ethical aspect of veterinary practice is not always taught in universities, making it difficult to conclude these cases [8,9]. Even when clinicians can address this issue, their professional responsibility forces them to advocate for both parties’ interests: the guardian and the animal’s health [10]. Moreover, the legal framework in different countries (e.g., Austria and Germany) permits euthanasia methods for a “good reason”. This means reasons owners can provide and might not necessarily be linked to medical or pathological conditions threatening the well-being of the animal.

For this reason, this review aims to explore the ethical and legal implications of euthanasia in canine patients. It will analyze the reasons that can justify euthanasia in animals with pain or terminal and chronic degenerative diseases, highlighting the importance of effective communication, ethical knowledge, and consideration of euthanasia as a multimodal resolution.

## 2. Search Strategy

**Figure 1:**
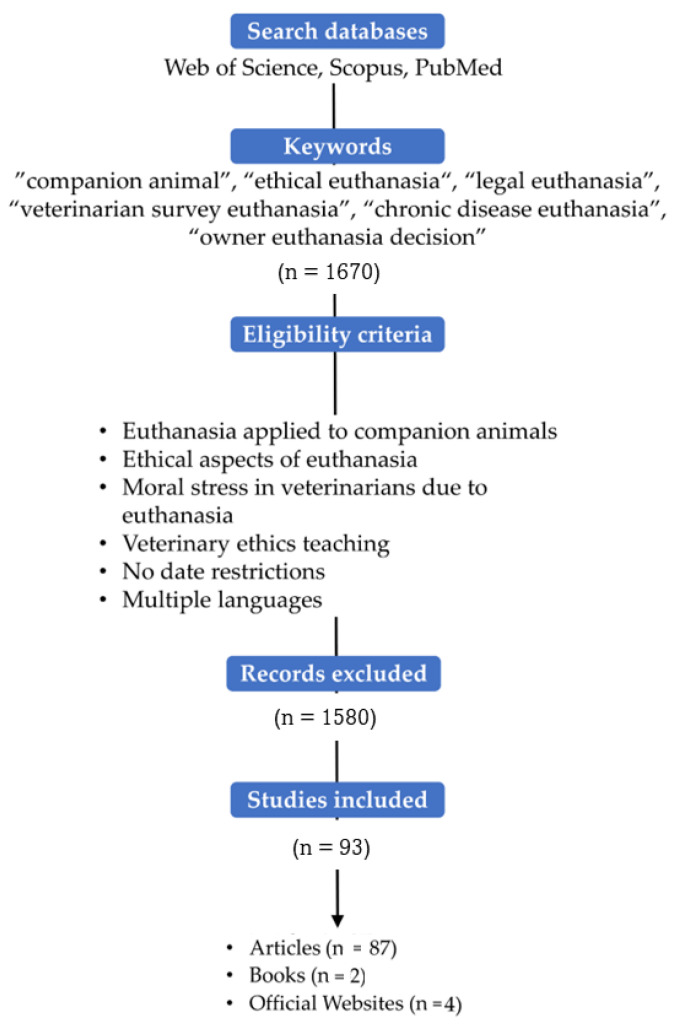
Diagram of the search strategy.

The literature search was conducted on Web of Science, Scopus, and PubMed, using the key concepts: “companion animal”, “ethical euthanasia”, “legal euthanasia”, “veterinarian survey euthanasia”, “chronic disease euthanasia”, and “owner euthanasia decision”. Figure 1 describes the overall search methodology, mentioning the inclusion of the studies where an ethical approach was assessed.

## 3. The Euthanasia Process as a Challenging Decision for Veterinarians

In veterinary medicine, euthanasia is considered a humane act to alleviate the animal’s suffering and pain [11]. Although it is considered a positive action to protect the welfare and integrity of animals, ending their life raises a complex ethical controversy [12]. One of these dilemmas is deciding from which point extending life may imply prolonging the pain [6,10,13], while respecting the owner’s decisions [10].

Some studies, such as Moses et al.’s [14], have shown that ethical conflicts are part of everyday practice and are challenging for veterinarians, where euthanasia of sick and healthy animals is one of these ethical responsibilities [15]. A survey of 889 North American veterinarians reported that 79% had obstacles identifying ethical conflicts, and more than 70% of the respondents considered that facing and resolving these issues causes moral, physical, and mental stress and anxiety [14,16,17]. It is a serious issue since it is recognized that facing the decision and medical responsibility to end the lives of companion animals causes high stress, as reported by Herzog et al. [18]. This emotional response is not only associated with the euthanasia process, but with factors that include the animal’s suffering, the guardian’s opinion, or the lack of improvement when treatment protocols cannot restore the animal’s health [18].

The limited teaching of veterinary ethics as an independent subject (e.g., in Latin American or European countries) and decision-making skills are suggested as reasons why practitioners find euthanasia challenging when it comes to complying with their obligations towards the animals, clients, and colleagues. In this context, Kipperman et al. [5] surveyed 284 veterinary students from four schools in the US to determine the benefits of teaching ethics to confront ethical dilemmas. Overall, 80% and 55% of the students indicated that the training in ethical theories helped them to identify and address these conflicts, respectively. Most respondents (54%) reported that ethical training helped them cope with moral stress. Both studies are proof that veterinarians can use ethics as a tool to identify if proceeding to euthanasia is the right choice, decide on the way to communicate it to the owner, and even face the moral challenges that euthanasia implies.

The role of the guardian is essential when discussing euthanasia in companion animals. In most cases, the suggestion for euthanasia comes from the owner for reasons beyond a solely medical aspect. These include a reluctance to address behavioral issues or economic constraints that add to the ethical and legal challenges for the veterinarian [19]. According to Passantino et al. [20], although these reasons may be relevant for the owner, they cannot be used alone to settle for euthanasia [21,22]. However, a survey performed on 12 dog guardians regarding the consideration of medical treatment or euthanasia of their pet due to age or chronic disease found that persons hesitated in deciding because there is no clear cut-off point to assume by themselves if their dog is in good health without acute clinical signs of an obvious disease. Therefore, the knowledge and guidance of the veterinarian are essential [23]. These authors’ findings indicate that the decision is morally challenging. However, training in ethical aspects could help to lessen the impact and help with the decision by considering multiple factors such as emotional and financial aspects.

Consequently, deciding on euthanasia is an emotionally and morally stressful process for the veterinarian due to the dilemma of recognizing the physical and physiological indicators of the animal’s health that could mark an appropriate endpoint. For this reason, ethical aspects, physical exams, laboratory tests, behavior-based scales, and an overall evaluation of the animal’s well-being must be considered to establish when the quality of life is inadequate [24]. Both the guardian and the professional must discuss all the available alternatives, and the benefits or consequences of the pet’s life.

## 4. Ethical Implications of Euthanasia in Companion Animals

An ethical perspective considers the different beliefs and values each individual assigns, resulting in multiple ways to approach the same subject [25], and simultaneously evaluates moral issues and the reasoning behind their views [26]. When considering euthanasia processes in companion animals, ethics contemplates notions such as when it is “right” or “wrong” to suggest the option to the owner according to the current state of the animal [7,27]. A clear example is end-of-life decision-making when treatment is no longer effective [25]. In this sense, the ethical basis would be to consider that all medical and financial alternatives have been used in the sick animal since client income is a strong predictor of euthanasia when guardians with financial constraints are less able to afford further treatment [28]. Table 1 summarizes the main reason for euthanasia in dogs according to different studies.

Another reason that might implicate occupational stress for veterinarians is those cases where dogs have behavioral problems, and owners suggest euthanasia due to their incapability to deal with or correct the issue. A hypothetical case of euthanizing a dog that attacked another non-human animal or child would end a healthy animal’s life. From a veterinarian’s perspective, the owners decision must be respected regardless of the animal’s health because refusing the procedure could increase the risks of another attack [35], or the risk of the owner deciding on less humane ways to deal with the animal. Yu et al. [36] analyzed the mortality rate of dogs between 1 and 3 years of age due to behavioral problems. They observed that 29.3% of 4341 dogs died in 3 years or less. Interestingly, only 11% referred the case to a specialist, only 5.9% attempted pharmaceutical therapy, and aggression was the main issue. Reiser et al. [37] also evaluated the association between dominance-related aggression and the risk of euthanasia in 110 dogs analyzed through logistic regression. In two models, severe aggression in response to benign dominance and body weight > 18.2 kg was associated with euthanasia.

When guardians request for euthanasia of an aggressive animal, the veterinarian should act as a mediator and assess whether the animal can be a candidate for rehabilitation programs, pharmacological treatment, or adoption [35]. An example of this is described by Benedetti et al. [38] in a case of an aggressive dog where sterilization, behavioral rehabilitation, and drug treatment with fluoxetine did not improve the dangerousness of the animal, and animal associations advocated for the dog in order to prevent euthanasia and maintain the behavioral treatment, regardless of the risk. In particular, these cases challenge the ethics of veterinary professionals because they carry moral, legal, and societal obligations in their decisions [39]. According to Tudor [40], any professional who finds themselves in this situation must act with ethical responsibility towards the patient, the client, and the general public, so an assessment of a history of aggression could help to guide whether the animal is a real threat to the public, or if teaching responsible ownership and even adoption are likely resources [41].

Another scenario with ethical dilemmas for the veterinarian is those animals with a chronic degenerative disease. For example, owners tend to consider euthanasia as an alternative in aging blind animals because, for them, the quality of life has deteriorated due to their disability. However, performing euthanasia on these animals without evidence of the diminished quality of life or any other generalized illness is controversial. Biondi et al. [42] mention that visual impairment is not always associated with a severe systemic disease that would threaten the quality of life in the long term. Therefore, communication with the owner is crucial to provide solutions, alternatives, and proper management depending on the case [43]. Although effective euthanasia communication with the guardian is essential for daily practice, it is one of the least prioritized skills in a veterinarian curriculum [44], but necessary to explain in which cases the health of an animal is threatened or will decline in a short period [45].

In the case of animals with cardiovascular, renal, hepatic, neurologic, and cancer diseases where the treatment is no longer providing adequate results, there are ethics questions regarding whether it is correct to extend the suffering of an animal, and until which point. A comparative study conducted by Pugliese et al. [34] evaluated the causes that led guardians to choose euthanasia in comparison to an unassisted death. They found that 40.7% and 50.8% of the cases were due to euthanasia and unassisted deaths, respectively. In addition, the main causalities associated with euthanasia were due to neoplastic (75.6%), degenerative (64.3%), and congenital (60%) diseases, and risk predictors of euthanasia included sex (female), age, and neoplasia. According to the Hippocratic oath that establishes “first do no harm”, the conclusion to postpone euthanasia in a terminally ill animal would mean prolonging their pain and suffering [3]. In these patients, an approach to evaluate, together with the owner, the current health state of the pet is by assessing the presence and severity of clinical signs and physiological markers. Authors such as Devi et al. [46] mention that in dogs with heart disease, a nocturnal cough is present in 2.55% of cases, while intolerance to exercise and hyporexia or anorexia in 1.82% of cases. On the other hand, in animals with chronic kidney disease, uremic syndrome is commonly reported with signs such as anorexia, muscle hypotrophy, increased creatinine, and plasmatic urea [47,48], as well as a decreased number of functional nephrons and an impaired glomerular filtration rate [49]. The accumulation of uremic compounds is present in the mucosa, with the typical canker sores in the oral mucosa and throughout the digestive tract causing acute pain in the animal [50,51]. According to the International Renal Interest Society (IRIS), therapeutic management criteria and disease progression need to be determined using markers such as symmetrical dimethylarginine (SDMA) for chronic kidney disease and glomerular filtration rate [52,53]. In canine studies with acute and chronic kidney injury, SDMA increased between 35 and 39.5 μg/dL [54], and is associated with illness progression and renal function decline [53].

A similar situation occurs in animals suffering from chronic pain due to cancer or osteoarthritis and the sensitization effect that constant proinflammatory cytokine and interleukin release produce in nociceptors, causing neuropathic pain [55,56,57]. Different evaluation scales, such as the Liverpool Osteoarthritis in Dogs [58] or health-related questionnaires to assess the quality of life [59], have been developed to assess the degree of impact and response to treatment. In these cases, there is an evident deterioration of the animal’s health due to the progress of the disease, and deciding to prolong the animal’s life could lead to dysthanasia (painful and agonic death) [41,60]. When considering patients with oncological diseases, prognostic indicators such as mitotic indices (e.g., argyrophilic nucleolar organizer regions, Ki-67, and proliferating cell nuclear antigen) [61], nucleotide polymorphisms in HER-2/neu, BRCA1, and P53 genes [62], and levels of E-cadherin expression [63] as markers of tumor grade and metastatic process in mast cell tumors, mammary cancer, and squamous cell carcinomas should be taken into account. In dogs with lymphoma, positive B-markers (CD20, CD21, CD79a, CD3, CD5, and PAX5) are used to diagnose and classify the tumors [64,65].

When facing terminal illness or diseases that progress with severe pain, euthanasia is the moral and appropriate option [66] when these signs and markers help to identify disease progression and health deterioration linked to survival time [67,68,69]. Therefore, physiological and blood parameters can guide the professional and the owner. However, an important aspect is that the guardian’s determination may be influenced by emotional attachment [70]. Schoen et al. [71] highlight the emotional bond and the personality of the owner as a strong predictor for euthanasia, since owners that feel a strong commitment are willing to spend more time with their companion animal regardless of the level of quality of time. Oyama et al. [67] reported the election of the quantity of life over quality of life of 201 owners of dogs with cardiomyopathy. In total, 86% of the respondents preferred survival time over quality of life, and 52% of the youngest owners were willing to trade six months of life. The above reflects that even factors such as age, sex, and experience with previous pets affect the decision to continue with the treatment or perform euthanasia [45]. Caregiver burden is another factor reported in surveys analyzing the prediction of the consideration of euthanasia [28].

If ethics contemplates what practices can be regarded as right and wrong in the medical profession, prolonging the suffering of an animal would not only mean acting against veterinarian principles, it could also negatively impact their mobility, hygiene, physiological functions, and mental health [72]. Some authors view the health decline and losing the ability to perform basic physiological functions as the outset of euthanasia [72,73]. The decline of companion animals’ welfare and quality of life is a topic that must be conveyed to the owners to reach agreements [74].

Consequently, although the owner´s desire must be considered during the euthanasia decision, the objective of the veterinarian is to protect the animal’s welfare and health. Effective customer communication must be considered during a critical decision to bring both owner and patient satisfaction.

## 5. Legal Implication for Euthanasia in Chronically Ill Animals

When considering the legal aspect of euthanasia in different countries, the aspect that all regulations have in common is that preventing and diminishing suffering is the main priority in the procedures and handling of animals [75], while preserving the quality of death. In this sense, since euthanasia implies a fast and painless induction of unconsciousness followed by death [19], surveys have shown that the quality of death is not only beneficial for animal welfare and the veterinarian but can also influence the grieving and acceptance process of the client in the last moments with their companion animal [76]. Most guardians have a strong bond with their pets and will accept treatment until they have exhausted all the alternatives [77]. When euthanasia is refused, the clinician needs to communicate the short-term progression of the disease and be mindful of how this can affect the moment when death is imminent (e.g., representing a traumatizing event for the client due to the animal’s suffering). This example is one of the major ethical and professional issues, since, under the law, companion animals are regarded as properties., However, recent works have stated that animals could be regarded as legal persons with the implication inside the legal system [78], and the client has the right to decline euthanasia and ask for advanced veterinary care and treatments to prolong the animal’s life as long as possible, sometimes neglecting the quality of death [79].

There is no consensus on a universal definition of “good death” because it can depends on the owner’s perspective, finances, culture, and previous experiences [80]; however, to preserve it, veterinarians must abide by the law to recognize and decide the cases where euthanasia is the right choice [19]. In countries such as Italy, Passantino et al. [20] mention that companion animal euthanasia is partially regulated by law and can be exclusively performed on seriously ill individuals. A key concept when legally referring to euthanasia is “deemed to be in the patient’s best interest”. This means that the decision should be based on a medical professional’s opinion, the individual’s values, and the available resources for the treatment [81]. Nonetheless, animals cannot vocalize opinions, and their “best interest” is directly marked by the owner and their knowledge about the disease, animal welfare, and other personal factors [42].

In the United States, the legislation in 42 states (e.g., Alabama, California, Colorado, Connecticut, Florida, Georgia, Maine, New Jersey, Wyoming, among others) justifies the euthanasia of an animal that has caused death or serious injuries, acknowledging them as a potential risk to the civilian population [82,83] and considering euthanasia lawful within the framework of public safety [21]. The veterinarian’s refusal to perform euthanasia would possibly bring legal or disciplinary consequences because the legislation considers the interests of both the owner of the animal and the general public [19]. In Latin America, veterinarians receive training courses where euthanasia is one subject among other animal welfare-related regulations [84].

Regarding animals with a chronic-degenerative disease, legislation in countries such as Germany and Austria establishes that a good reason is required to end an animal’s life [85]. However, the term “good reason” is vague, subjective, and broad, and can vary depending on the understanding of the people towards their companion animal [19]. In this sense, the human–animal interactions and the individual perception of what is good/bad for the animal might interfere not only with the veterinary profession but with the laws by incorporating their meaning into the legislation [86]. To amend this, in the United States, the law states that euthanasia can be an option for sick, seriously injured, and dangerous animals only if the procedure is recommended by a licensed veterinarian [83,87,88].

Persson et al. [19] argue that euthanasia responds to various social and economic factors with sentimental value for the guardians. An example would be a dog diagnosed with cancer known to produce severe pain. In this case, the person responsible for the animal can exercise their right to resort to euthanasia since it is in “the best interest of the pet” without appealing to pharmacological treatment, and no illegality would be established. However, from an ethical perspective, euthanasia should be the clinician’s and the owner’s last resort only after multimodal treatments have been tested without favorable outcomes or due to limited financial resources [6,19].

When the guardian refuses to resort to euthanasia due to a human emotional response (e.g., grief), according to the Animal Welfare Act, the owner breaches the law by allowing and prolonging the suffering of the companion animal. The Animal Welfare Act established in England, Scotland, and Wales mentions that “…if a veterinarian certifies that the condition of the animal is such that in its interest it should be destroyed, he must act without the consent of the owner…”, and this is to alleviate the suffering of companion animals [89]. Nonetheless, the ethical weight of the respect for animal life and the owner’s decision is a prevalent challenge for veterinarians.

The discussed information shows that legislation towards euthanasia in pets is always driven by animal welfare. Although different countries legislate in favor of animal welfare, the reason that can be considered appropriate for euthanasia is too broad, involving non-medical social factors or socio-economical aspects [90]. Therefore, it is necessary to establish terms and state rules regarding euthanasia in animals with chronic-degenerative diseases since most of this act is justified for sentimental reasons, and it is not clear what reasons are acceptable.

## 6. Perspectives

Since euthanasia is a procedure that most veterinarians will perform at least once in daily practice, it is essential to recognize that this implies moral and social stress even when the technique is ethically indicated in terminally ill patients [91]. One of the strategies that veterinary medicine could have to face this challenge is to reinforce ethical teaching in universities so that colleagues can have the tools to decide whether the decision for euthanasia is the appropriate one, and have the skills to effectively communicate and discuss euthanasia with the owners [92].

The emotional burden that euthanasia represents for the clinician and the guardian is another critical topic that needs to be broadly taught in veterinary medicine. While it might not be the direct role of veterinarians to treat this problem, studies by Spitznagel et al. [93] have shown that distress in clients can impact their perspective towards euthanasia and they can hastily decide before considering previous treatments for their pet. The way the owner perceives animal welfare and their level of empathy is also an interesting research field where high levels of empathy have been associated with euthanasia decisions at later stages of the disease [94], and emotional support provided by veterinarians is proposed as a compassionate way to conduct euthanasia [95].

The legislation on euthanasia in companion animals has its limitations. The close bond of animals with their owners, regarding them as other family members, is an excellent opportunity to adjust the laws to events that were not contemplated.

One of these events could be incorporating the emotional aspect of the euthanasia decision and other cases when the animal is healthy. However, the guardian decides for personal and economic reasons. For example, although legally, the right of ownership lets clients propose euthanasia on a healthy dog because of excessive barking or moving to a different house that does not allow pets, this right does not obligate veterinarians to proceed to euthanasia when there is no justifiable reason [96]. These cases need to be considered inside regulations to give clinicians more space to discuss and propose alternatives to euthanasia. On the contrary, when the owner refuses euthanasia due to emotional attachment, an upgraded legal framework could somewhat clearly state until which point, from a physiological point of view, it is ethically correct to continue with a treatment. Certain biomarkers could be added as a mandatory evaluation to establish disease progression and deterioration of quality of life to aid in this objective.

## 7. Conclusions

The decision to choose euthanasia in animals with chronic-degenerative diseases is a morally stressful event for the veterinarian. Socioeconomic, emotional, and pathological factors influence the decision on euthanasia. However, from a veterinarian’s perspective, ethical consideration has proven to be the most reliable option for dealing with stressful situations and providing guidance to owners. Ethical teaching can contribute to lessening the burden of clinicians and distinguishing between right and wrong in morally questionable cases.

Finally, the current laws are limited regarding the care of animals with chronic-degenerative diseases. Most of them provide vague terms that do not consider the potentially harmful factors of the disease that cause chronic pain. Therefore, the current legislation should undergo significant reform, leading to actions and strategies of the veterinarian in favor of the animal’s health.

## Figures and Tables

**Table 1:** Main causes of euthanasia according to different studies.

Author	Country	Demography	Cause	Count (%)
Damián et al. [29]	Uruguay	100 veterinarians	Neoplasia	34
			Age	30
			Infections	14
			Kidney disease	12
			Trauma	7
			Others	3
De Souza et al. [30]	Brazil	39 dogs 23 males 16 females	Distemper	24.4
			Spine fractures	14.6
			Neoplasia	12.2
			Renal insufficiency	4.9
Freitas et al. [31]	Brazil	651 dog necropsies	Neoplasia	30.41
			Infectious and parasitic disease	25.65
			Degenerative disease	12.90
			Inconclusive	12.29
			Physical agents	9.52
			Metabolic disease	3.38
			Poisoning	1.54
			Developmental disorder	1.08
Moore et al. [32]	United States	927 military working dogs Mean age 10.06 years Belgian shepherd German shepherd 11 other breeds	Degenerative joint disease	19.2
			Neoplasia	18.3
			Spinal cord disease	15.6
			Geriatric	14.1
			Cardiac disease	3.7
			Behavioral disorder	2.0
			Urogenital disease	1.8
			Ophthalmologic disease	1.2
Pegram et al. [33]	United Kingdom	26,676 dogs Median age 12.1 years Median weight 18.4 kg	Neoplasia	11.6
			Collapsed	11.7
			Non-defined mass growth	8.0
			Behavioral disorder	7.1
			Musculoskeletal disorder	5.9
			Kidney disease	3.4
			Traumatic injury	1.1
Pugliese et al. [34]	Italy	125 dogs Median age 96 months	Neoplasia	75.6
			Degenerative disease	64.3
			Congenital disease	60
			Vascular disease	40
			Toxic	38.5
			Inflammatory	34.9
			Traumatic	18.2
